# MicroRNAs and hepatitis C virus: Toward the end of miR-122 supremacy

**DOI:** 10.1186/1743-422X-9-109

**Published:** 2012-06-12

**Authors:** Thomas Walter Hoffmann, Duverlie Gilles, Bengrine Abderrahmane

**Affiliations:** 1 EA4294 Unité de Virologie Clinique et Fondamentale, Université de Picardie Jules Verne, UFR de Médecine et de Pharmacie, 3 rue des Louvels, 80036, Amiens Cedex, France; 2 Laboratoire de Virologie, Centre Hospitalier Universitaire d’Amiens, Avenue René Laennec, 80480, Salouël, France; 3 Biobanque de Picardie, Centre Hospitalier Universitaire d’Amiens, Avenue René Laennec, 80480, Salouël, France

**Keywords:** MicroRNAs, Hepatitis C virus, Virus-host interactions, New therapeutic targets

## Abstract

The most common etiologic agents causing chronic hepatitis are hepatitis C and B viruses (HCV and HBV, respectively). Chronic infection caused by HCV is considered one of the major causative agents of liver cirrhosis and hepatocellular carcinoma worldwide. In combination with the increasing rate of new HCV infections, the lack of a current vaccine and/or an effective treatment for this virus continues to be a major public health challenge. The development of new treatments requires a better understanding of the virus and its interaction with the different components of the host cell. MicroRNAs (miRNAs) are small non-coding RNAs functioning as negative regulators of gene expression and represent an interesting lead to study HCV infection and to identify new therapeutic targets. Until now, microRNA-122 (miR-122) and its implication in HCV infection have been the focus of different published studies and reviews. Here we will review recent advances in the relationship between HCV infection and miRNAs, showing that some of them emerge in publications as challengers against the supremacy of miR-122.

## Review

**Figure 1:**
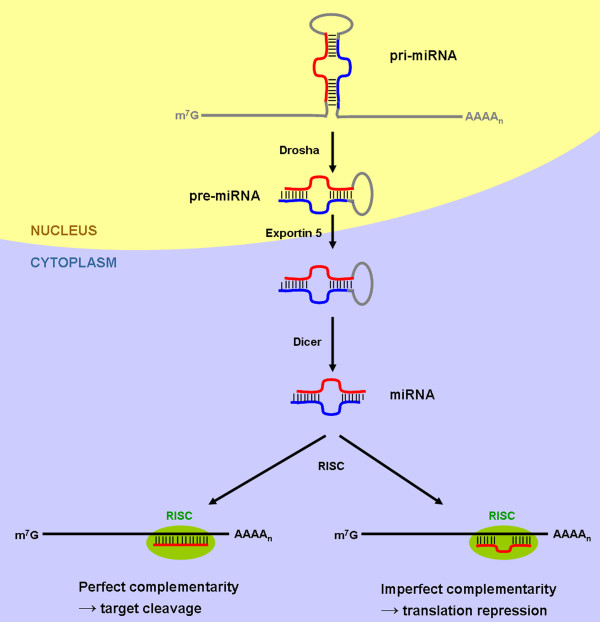
**Canonical miRNA biogenesis and function.** miRNAs are transcribed as capped and polyadenylated primary transcripts (pri-miRNAs) containing a hairpin structure that is excised by the endonuclease Drosha. This liberated stem-loop (pre-miRNA) is transported to the cytoplasm by exportin 5 to be processed by the Dicer endonuclease. RNA-induced silencing complex (RISC) selectively handles one strand of the resulting duplex and triggers target mRNA silencing through cleavage or translational repression

Nearly 20 years after their discovery, we now know that microRNAs (miRNAs) modulate gene expression networks. These small non-coding RNAs (around 22 nucleotides) have been shown to function as regulators of gene expression. First discovered in *Caenorhabditis elegans*[1], miRNAs have quickly been considered a fundamental component of the regulatory system of gene expression and have been estimated to control at least one third of eukaryotic cell genes. miRNAs are transcribed as long primary transcripts (pri-miRNAs) and trimmed into approximately 70 nucleotide stem–loop precursors (pre-miRNAs), most of the time by Drosha in the nucleus [2]. Pre-miRNAs get exported to the cytoplasm by Exportin-5 and processed by Dicer to generate mature miRNAs [3,4]. The mature miRNAs then direct the binding of Argonaute-containing protein complexes (RISC, for RNA-induced silencing complex) on specific messenger RNA (mRNA) targets to promote their degradation or to affect their translation ([3] and Figure 1). The 5' nucleotides, 2–7 of the mature miRNA, are called the seed sequence and have the strongest influence for target selection. miRNAs that share the same seed sequence are grouped into families, however they may not have the same targets [5]. One miRNA has the ability to target many different sites on the same mRNA or on many different mRNAs.

Today, more than 1500 miRNAs have been identified in the human genome (miRBase release 18 [6-8]), and their biological functions are under investigation. Despite the important advances accomplished to elucidate their role in regulating different physiological and pathological processes, our understanding of their implications in response to viral infection (hepatitis C virus for instance) is in its early stages.

Hepatitis C virus (HCV) is a small enveloped positive-strand RNA virus of the Flaviviridae family, classified in the genus Hepacivirus. It infects around 3% of the world population and it is the most common etiologic agent underlying chronic hepatitis with hepatitis B virus (HBV). Chronic HCV leads to cirrhosis of the liver and ultimately to the development of hepatocellular carcinoma (HCC) [9]. Globally, 31.1% of HCC cases are caused by HCV infection, whereas HBV is responsible for 54.4% of HCC cases [10]. The prevalence of HCC, fifth in term of frequency and third in term of mortality within cancers, demonstrates the enormous threat posed by HCV infection to the worldwide public health. Moreover, anti-HCV treatments show irregular efficiency between viral genotypes and the ethnic origin of the infected patients. One example of this irregularity is demonstrated in patients who are infected with the type 1 genotype. They typically only show 50% effectiveness when treated with pegylated interferon (pegIFN) and ribavirin [11]. The rate of sustained viral response (SVR) to treatment is improved to 70% when antiproteases are associated with this standard regimen [12]. Other combinations with new molecules are expected to promote HCV eradication in almost all patients regardless of the HCV genotype.

Recent advancements in understanding virus-host interactions uncovered multiple host factors used by HCV to infect cells [13]. As a major factor in controlling different cellular processes, miRNAs represent an interesting field of investigation regarding HCV infection and replication. Furthermore, they may represent new targets for the development of antiviral therapeutics. The work of Yang *et al.* provides a proof of concept for the potential use of these molecules. Indeed, these authors showed that HCV replication *in vitro* and in a mouse model was inhibited when 5 artificial anti-HCV miRNAs, cloned in the miR-17-92 cluster and delivered by an adenovirus vector, are expressed [14]. These 5 artificial miRNAs reduced the replication of cell culture-propagated HCV (HCVcc) by 98% and resulted in 93% HCV gene silencing in mouse livers without causing any hepatocellular toxicity. However, these results should be taken with caution as it has also been shown that over-expression of certain pre-miRNA-like molecules in the liver is lethal in mice [15]. Recently, Berezhna *et al.* reported that replication or suppression of HCV RNA depends on the interaction of Ago2 protein (member of RISC complex) with miRNA or small interfering RNA (siRNA) and its partitioning to different cell compartments [16]. This finding is an additional proof in favor of the therapeutic potential of miRNAs.

Since the first publication reporting the positive role of miR-122 in HCV replication [17], this liver-specific miRNA has been a focus of numerous research projects investigating the liver and HCV interaction. It has 4 binding sites in the HCV genome [18], it is implicated in regulation of different metabolic pathways in liver cells (cholesterol metabolism) [19] and it represents 50% to 70% of all the miRNAs expressed in the liver [20-22]. An anti-HCV treatment based on miR-122 inhibition by antisense oligonucleotides has been tested on chimpanzees and provided very promising results [23]. The same anti-miR-122 molecule is also the first miRNA-targeting treatment to enter into human clinical trials (currently in phase 2) to treat patients infected with HCV (miravirsen, Santaris Pharma A/S). Even though miR-122 has been the most studied miRNA in HCV infection and the clinical trials using anti-miR-122 have shown promising results against HCV, this strategy against miR-122 might have some negative impacts on the hepatocytes’ metabolism. Therefore, other miRNAs may also play an important role in HCV-host interactions and be good candidates for the fight against HCV. This is the reason why several research teams around the world are interested in bringing to light the whole “miRNome” and uncover the role played by other miRNAs in controlling such interactions. Randall *et al.* reported a systematic RNA interference (RNAi) screening study on the human hepatoma cell line Huh-7.5 [24]. In this work they targeted 62 cellular genes, using siRNAs thought to play a role in HCV infection, and demonstrated that RNAi targeted 26 of these genes and reduced viral production by at least 3 fold. Some of these siRNAs targeted Dicer or other components of the RNAi pathway, therefore confirming the close interaction between HCV and the miRNA machinery. Other studies reported specific miRNAs altering HCV infection and/or being altered by the presence of the virus. It is reasonable to assume that some of these miRNAs may have a promising potential as therapeutic targets, such as miR-196b, miR-199a-3p and miR-29 that inhibit HCV replication in several models [25-27]. Despite this promising potential, these miRNAs are not as intensively studied as miR-122 [28-30].

In this review, we will therefore discuss recent advances in the relationship between HCV infection and miRNAs and show that some of them might be as important as miR-122 in the fight against HCV.

### miR-196b

The mir-196 gene family contains 3 members (mir-196a-1, -196a-2, and -196b) and are found in the homeobox clusters of vertebrates [31]. Two mature miRNA, miR-196a and miR-196b, differing by only one nucleotide, are products of these 3 genes [31]. miR-196 was first reported to have extensive and evolutionarily conserved complementarity to homeobox clusters and to regulate the expression of homeobox genes such as HoxB8 [32]. In addition to its role in development, many studies on miR-196 reported its role in several cancers (for review, see [31]). The increased expression of miR-196b in HCC when compared with normal livers [33] suggests that this miRNA may have an oncogenic role in HCC.

It has been reported that miR-196b has a target site in the NS5A coding region of HCV RNA (Table 1 and [27]). The expression of miR-196b and eight other miRNAs predicted to target HCV RNA is modulated upon interferon (IFN) β treatment of primary mouse hepatocytes and Huh7 cells [27]. Because IFNβ significantly affects the innate anti-HCV response, the authors tested the impact of these IFNβ-induced miRNAs on HCV replication. Only 5 miRNAs decreased HCV replication, including miR-196b and an antisense oligonucleotide against miR-122. The decrease of HCV replication ranged from 50% to 80% (60% for miR-196b). Later, Scagnolari *et al.* confirmed the increase of miR-196b expression by the IFN pathway in human peripheral blood mononuclear cells (PBMC) of healthy individuals [34].

**Table 1:** Summary of the matches implied in HCV inhibition by 3 microRNAs considered as potential therapeutic targets

**microRNA**	**Inhibition of HCV infection by**	
	**Matches on HCV RNA**	**Matches on host targets**
miR-196b	NS5A coding region (6680) [27]	Bach1 3’UTR (2286 and 2166) [35]
miR-199a-3p	5’UTR IRES (60) [26]	NR
miR-29	NR	NR

The numbers in brackets indicate the first nucleotide of the binding sites of the miRNAs.

UTR: untranslated region; IRES: internal ribosome entry site; NR: non reported.

**Figure 2:**
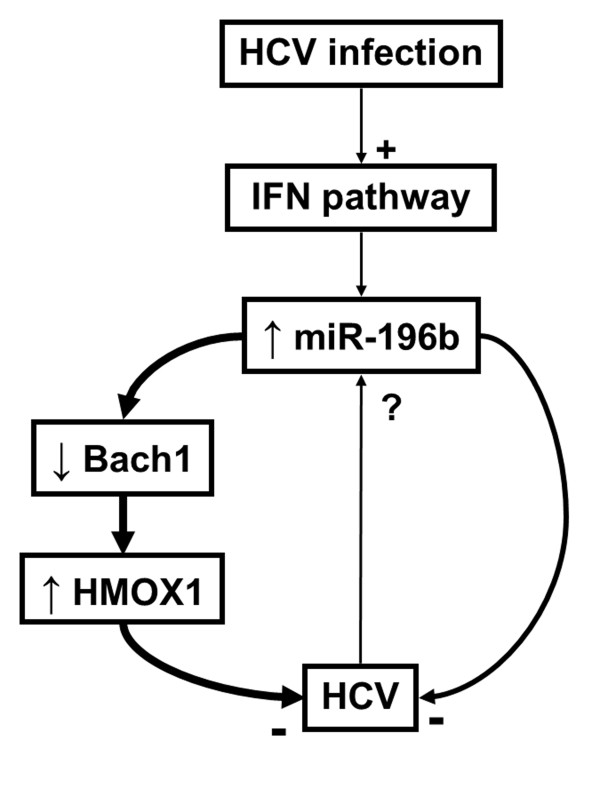
**miR-196b and hepatitis C virus infection.** After activation by hepatitis C virus (HCV) infection, interferon (IFN) pathway stimulates the expression of miR-196b. This miRNA inhibits HCV replication either directly via a target site on the HCV RNA or indirectly via Bach1 down-regulation and heme oxygenase-1 (HMOX1) up-regulation. The indirect pathway seems to be predominant. How HCV responds to miR-196b targeting remains to be fully investigated

The mechanisms involved in the inhibition of HCV replication by miR-196b were later revealed by Hou *et al.* (Figure 2). In addition to the direct targeting of the HCV RNA, Bach1, a repressor of the anti-oxidative and anti-inflammatory heme oxygenase 1 (HMOX1), is also a direct target of miR-196b [35]. In this study, this miRNA was able to inhibit HCV expression in replicon cell lines (around 50% of inhibition) directly by targeting HCV RNA and indirectly by increasing HMOX1 expression. Evidence suggests that the importance of Bach1 targeting is predominant over direct HCV targeting, however the mechanisms of HCV inhibition by HMOX1 remain unknown. As HCV infection causes oxidative stress and miR-196b inversely modulates HCV replication and HMOX1 expression, overexpression of miR-196b appears as a potential strategy to protect against liver injury during chronic hepatitis. Interestingly, miR-122 has also been linked to the down-regulation of HMOX1 [36], which means that the overexpression of miR-196b could be even more efficient if associated with inhibition of miR-122 [36].

It is unknown whether HCV has evolved to counteract the effect of this antiviral miRNA, although some studies suggest that such a mechanism might exist. Indeed, the expression of miR-196a and miR-196b decreases upon HCV transfection of HepG2 cells as well as in PBMC of chronic hepatitis C patients [34,37]. This decrease could be linked to the inhibition of IFN pathway by the virus, since in PBMCs from chronic hepatitis C patient the expression of miR-196b does not change after IFNα treatment, whereas it is increased in PBMCs from healthy controls [34]. However these results remain to be formally proven, as it has been shown in one study that miR-196b and miR-196a seem to be respectively increased and decreased in HCV replicon-containing cell lines [38]. Furthermore, Grek *et al*. reported in another study that miR-196b was increased in PBMC replicating HCV [39]. HCV infection and replication in PBMCs still remains controversial, but these results suggest that the interactions between miR-196b and HCV infection are complex. These discordances might also be evidence of the limitations of cell lines and replicons against primary cells and complete virus cycle, respectively. In the light of these data, it could be hypothesized that miR-196b expression is (i) induced by the IFN pathway in cells replicating HCV and (ii) decreased by the pro-viral mechanisms, although this has yet to be verified.

### miR-199a-3p

According to miRBase release 18 [6-8], the miR-199 family is derived from 3 genes: mir-199a-1, -199a-2 and -199b. In terms of read number, the functional mature miRNA appear to be the 3p form for both miR-199a and miR-199b. Several studies reported an anti-tumoral role for miR-199a-3p, which targets the proliferation and migration/invasion signaling pathways in different cancers, especially HCC [22,40-42]. Others suggested that miR-199a-3p anti-tumoral activity is not associated with a specific cancer [43]. miR-199a-3p has also been associated with liver fibrosis progression [44] and alcoholic or nonalcoholic liver injuries [45].

**Figure 3:**
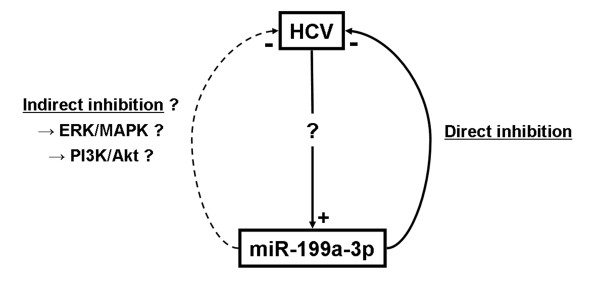
**miR-199a-3p and hepatitis C virus infection.** miR-199a-3p shows antiviral activity by directly interacting with HCV RNA and by inhibiting its replication, which increases the chance of a sustained viral response to antiviral treatment. miR-199a-3p might also exert an indirect inhibition of HCV via signaling pathways, as it were reported for other viruses. How this relates to the fact that HCV stimulates miR-199a-3p expression remains to be shown

In the context of viral infection, miR-199a-3p has been identified as a broadly active antiviral miRNA against members of all three herpesvirus subfamilies and the positive sense RNA virus Semliki Forest virus [46]. In this study, miR-199a-3p modulated the signaling pathways involved in viral replication such as PI3K/AKT and ERK/MAPK. The identification of miR-199a-3p binding sites in the 5’UTR IRES of HCV RNA (genotypes 1b and 2a) [26] suggested a direct interaction between these molecules (Table 1). Furthermore, overexpression of miR-199a-3p has an 80-90% inhibitory effect on virus replication, whereas viral replication and protein expression are promoted by the suppression of miR-199a-3p [26]. In the same study, Murakami *et al.* showed that miR-199a-3p induced viral RNA accumulation in the RISC complex as a consequence of direct binding to the 5’UTR IRES and was independent from the IFN pathway. Later, the same researchers showed that increased miR-199a-3p expression (with 34 other miRNAs) was associated with SVR in chronic hepatitis C patients when compared with non-SVR [47]. However, it remains to be investigated whether this miRNA inhibits HCV replication by direct binding to the viral RNA and/or indirectly via the negative regulation of cellular factors. It is worth noting that HBV replication is also inhibited by miR-199a-3p, as a consequence of direct interaction of the miRNA and the HBV coding region [48]. According to these pieces of evidence, the miR-199a-3p mechanism of action could be summarized as in Figure 3. They also suggest that this miRNA plays a major role in the hepatic antiviral response.

With such a strong effect on HCV replication, we could assume that miR-199a-3p expression is modified during HCV infection. In the numerous studies reporting miRNA expression profiles in HCV-infected cells or tissues, only two demonstrated an impact on miR-199a-3p by showing that the expression of this miRNA was increased during HCV infection [49,50]. It should also be noted that this miRNA is only weakly expressed in the liver [26], which means that further investigations are needed to fully assess the significance of the miR-199a-3p antiviral effect against HCV, as well as how HCV infection modulates the expression of this miRNA.

### miR-29

The miR-29 family comprises 3 members: miR-29a, -29b, and -29c, which are expressed from 4 genes, mir-29a, -29b-1, -29b-2 and 29c (miRBase release 18 [6-8]). To date miR-29 members have been implicated in the fibrosis of different organs such as the liver [51], kidney [52], heart [53] and lung [54]. Furthermore, miR-29 was also associated with IFNγ production [55]. These miRNAs appear to have tumor suppressor activity in leukemia [56] and HCC [57,58], as well as an antiviral activity against HBV [57].

**Figure 4:**
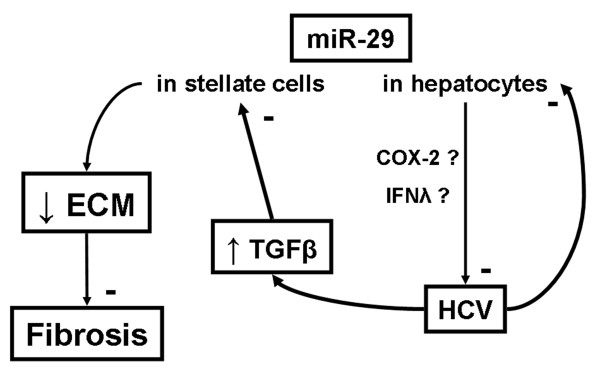
**miR-29 and hepatitis C virus infection.** miR-29 inhibits hepatitis C virus (HCV) replication in hepatocytes, maybe via cyclooxygenase-2 (COX-2) and IFN lambda (IFNλ), while at the same time HCV also inhibits miR-29 expression in hepatocytes to increase its replication. In hepatic stellate cells, miR-29 regulates the synthesis of the extracellular matrix (ECM), thus it inhibits fibrosis. HCV infection increases injury-associated TGFβ production, which inhibits miR-29 expression in stellate cells. This deregulates ECM synthesis and leads to fibrosis

The relationship between HCV infection and miR-29 family expression in hepatocytes and hepatic stellate cells (HSC) has been recently reported [25]. The analysis of miRNA profiles of HCVcc-infected Huh-7.5 cells and livers from chronic hepatitis C patients showed a 2-fold decrease of the 3 members of the miR-29 family compared to controls. The activation of HSCs also leads to the down-regulation of miR-29. Moreover, the same study showed that over expression of miR-29 in infected Huh-7.5 cells resulted in a 70% decrease of HCV replication. The over expression of miR-29 in HSCs inhibited their proliferation and their collagen production. Currently the mechanism explaining how HCV infection triggers the down-regulation of miR-29 in hepatocytes is unknown. It can be hypothesized that in HSCs the miR-29 down regulation is activated by liver injury-induced TGFβ along with the increased extracellular matrix production, which ultimately leads to fibrosis (Figure 4). Recently, the activation of cyclooxygenase-2 and IFN lambda production have been reported as mechanisms involved in the miR-29 antiviral activity against influenza virus [59]. This pathway has yet to be explored regarding HCV infection.

Others have documented the link between miR-29 family members and HCV infection. These studies showed the expression of miR-29a decreases in HCVcc-infected Huh-7.5 cells [60], and miR-29c is down regulated both in livers of patients presenting viral hepatitis (HCV or HBV) [61] and in livers presenting HCV-associated HCC [62]. Furthermore, in livers from chronic hepatitis C patients presenting SVR, miR-29a, -29b and -29c levels increased when compared to livers of patients without SVR [47]. This finding suggests that the miR-29 family (probably in combination with other miRNAs) could be a useful biomarker when monitoring the response to anti-HCV treatments.

Altogether there is a growing body of evidence suggesting the importance of the miR-29 family’s role in the cells’ defense against HCV and a treatment with miR-29 might inhibit HCV while reducing fibrosis.

### Other promising miRNAs

**Figure 5:**
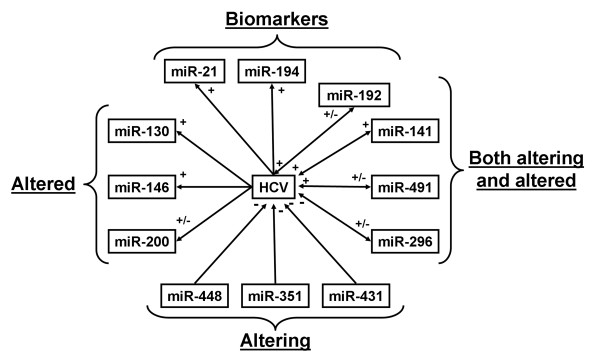
**Other promising miRNAs associated with hepatitis C virus infection.** Some miRNAs are altered by hepatitis C virus (HCV) infection and appear as potential biomarkers, or are simply altered by the virus. Others are reported as altering HCV replication. Four miRNAs are both altered by HCV and alter its replication

Several other miRNAs have been recently reported as associated with HCV infection, either because they altered viral replication and/or were altered by it (Figure 5). For instance, the expression of miR-141 is increased in HCV-transfected primary human hepatocytes when cocultured with a rat hepatic stellate cell line. Also observed was stimulated virus replication and production [49]. According to the same study, miR-141-mediated HCV stimulation involves the direct inhibition of the tumor-suppressor DLC-1.

By inhibiting the PI3K/Akt pathway, miR-491 stimulates HCV replication in replicon or HCVcc-infected Huh-7 cells [63]. This inhibition does not depend on the viral genotype, cell proliferation or HCV IRES activity. In HCV-infected Huh7 cells, miR-491 expression is increased during the first 4 days post-infection (up to 12 fold) [60], before going down after the fifth day [63]. Moreover, it is established that miR-491 also induces apoptosis [64]. These data suggest that miR-491 is involved in the early steps of HCV infection followed by a decrease in its expression, which might favor the development of HCC. Other interesting findings were reported by Ishida *et al.*, who demonstrated a stimulatory effect of miR-192 and −215 on HCV replication [63].

Meanwhile, the induction of miR-296 by IFNβ directly inhibits HCV replication in infected Huh7 cells by directly targeting the viral RNA [27]. Furthermore, the expression of miR-296 is decreased in the liver and increased in PBMCs of HCV-infected patients [34,50]. These findings suggest an unknown and complex set of mechanisms and interactions between HCV and miR-296.

As for miR-196b, the expression of miR-351, -431 and −448 is induced by IFNβ, thus these miRNAs may possess an inhibitory effect on HCV replication through direct targeting of the viral RNA [27]. The expression of miR-130, -146, -200, -192 and −194 is modified by HCV infection in several models as reported by different studies [37,38,49,50,60,63,65]. Interestingly, miR-192 and −194 are liver-specific miRNAs [66,67] and are associated with SVR in chronic hepatitis C patients [47]. Finally, miR-21, which is associated with liver regeneration [68] and HCC [69], is increased in HCV-infected livers and Huh-7.5 cells [50,70]. At least two studies have showed a correlation between miR-21 increased expression and (i) SVR [47], (ii) viral load, (iii) fibrosis (via inhibition of SMAD7), and (iv) level of transaminases in serum [70].

All these miRNAs represent promising targets for antiviral treatments and as potential biomarkers of HCV infection and its outcome. Further investigations will be required to fully characterize their cellular targets and their modes of action.

## Conclusions

There are now numerous pieces of evidence demonstrating the therapeutic potential of miRNAs during chronic HCV infection. Currently, miR-122 is the only miRNA tested in therapeutic clinical trials designed to treat HCV-infected chimpanzees [23] or human (Santaris, phase 2). Other miRNAs, especially miR-196b, -199a-3p and the miR-29 family, may possibly emerge as very promising targets for anti-HCV treatments. Based on the combination of all these miRNAs a new anti-HCV therapy could be proposed as follows: anti-miR-122, and mimics for miR-196b, miR-199a-3p and miR-29. This combination could also be used in conjunction with standard treatments to increase their efficiency. Such a protocol might have the advantage of preserving patients from HCV replication and from HCV-mediated injuries.

Due to miRNAs having multiple targets and their ability regulate different signaling pathways, it will be important to fully characterize their mechanisms of action and their cellular targets in order to avoid any negative patient outcomes. To date no serious adverse effect of miR-122 inhibition has been observed in chimpanzees [23] or healthy volunteers (Santaris, phase 2), however long term studies will need to be completed to ensure that over-expressed or blocked miRNAs don’t cause more damage than they prevent.

## Competing interests

All authors declare that they have no competing interests.
