# Correction: MicroRNAs and hepatitis C virus: toward the end of miR-122 supremacy

**DOI:** 10.1186/1743-422X-10-59

**Published:** 2013-02-15

**Authors:** Thomas W Hoffmann, Gilles Duverlie, Abderrahmane Bengrine

**Affiliations:** 1 EA4294 Unité de Virologie Clinique et Fondamentale, Université de Picardie Jules Verne, UFR de Médecine et de Pharmacie, 3 rue des Louvels, 80036 Amiens Cedex, France; 2 Laboratoire de Virologie, Centre Hospitalier Universitaire d’Amiens, Avenue René Laennec, 80480 Salouël, France; 3 Biobanque de Picardie, Centre Hospitalier Universitaire d’Amiens, Avenue René Laennec, 80480 Salouël, France

## Correction

After publication of this work [1], we noted that two of the authors were incorrectly named. The author list for this article should read Thomas W Hoffmann, Gilles Duverlie and Abderrahmane Bengrine.

## Competing interest

All authors declare that they have no competing interests.

## Authors’ contributions

TWH read the articles and gathered information cited in this review. GD revised the manuscript. TWH and AB drafted the manuscript. All authors read and approved the final manuscript.
